# Corneal biomechanical properties in myopic eyes evaluated via Scheimpflug imaging

**DOI:** 10.1186/s12886-020-01530-w

**Published:** 2020-07-11

**Authors:** A-Yong Yu, Hui Shao, Anpeng Pan, Qinmei Wang, Zixu Huang, Benhao Song, Colm McAlinden, Jinhai Huang, Sisi Chen

**Affiliations:** 1 School of Ophthalmology and Optometry, Wenzhou Medical University, Wenzhou, Zhejiang, China; 2 Key Laboratory of Vision Science, Ministry of Health Peoples Republic of China, Wenzhou, Zhejiang, China; 3 Department of Ophthalmology, Singleton Hospital, Swansea Bay University Health Board, Swansea, UK; 4 Eye Hospital of Wenzhou Medical University, 270 West Xueyuan Road, Wenzhou, Zhejiang, 325027 China

**Keywords:** Corneal biomechanics, Scheimpflug, Corvis ST, Myopia

## Abstract

**Background:**

To investigate the biomechanical properties of the cornea in myopic eyes using corneal visualization Scheimpflug technology (Corvis ST). The relationships between the biomechanical properties of the cornea and the degree of myopia were also investigated.

**Methods:**

265 eyes of 265 subjects were included. Based on spherical equivalent (SE) in diopters (D), participants were divided into four groups: low myopia/control (SE: − 0.50 to − 3.00D), moderate myopia (SE: − 3.00 to − 6.00D), high myopia (SE: − 6.00 to − 10.00D) and severe myopia (SE greater than − 10.00D). Axial length (AL), anterior segment parameters, and corneal biomechanical properties were obtained with the Lenstar LS900, Pentacam HR and Corvis ST, respectively.

**Results:**

Mean (±SD) SE was − 7.29 ± 4.31D (range: − 0.63 to − 25.75D). Mean AL was 26.31 ± 1.82 mm (range: 21.87 to 31.94 mm). Significant differences were detected within the four groups in terms of six corneal biomechanical parameters: deformation amplitude (DA), time from start until second applanation (A2-time), length of flattened cornea at the second applanation (A2-length), corneal velocity during the first and second applanation (A2-velocity), time from start to highest concavity (HC-time), and central curvature at highest concavity (HC radius). AL was positively associated with DA whereas negatively associated with A1-velocity and A2-length. SE was positively associated with A2-time, HC-time and A2-velocity, whereas negatively associated with DA. IOP was positively associated with four corneal biomechanical parameters and negatively associated with three parameters.

**Conclusions:**

Eyes with severe myopia showed greater DA, lesser A2 time, HC time, and faster A2-velocity compared to low to high myopia. This suggests the cornea becomes weaker and more deformable with elongation of axial length with corresponding increases in myopia. DA, A2-time and A2-velocity could be useful corneal biomechanical indicators in patients with myopia.

## Background

Myopia is the most common vision condition, affecting 70–80% of the population in many East Asian countries [1]. High myopia in Taiwan increased from 26% in 1988 to 40% in 2005 [2]. Brien et al. [3] estimated that myopia and high myopia will influence nearly 5 billion people and 1 billion people, respectively, by 2050. More importantly, a series of complications such as glaucoma, cataract, myopic retinal degeneration and retinal detachment are associated with high myopia, some of which may eventually lead to blindness [4, 5]. High myopia is characterized by scleral thinning and a sclera that is elastic and deformable [6–8]. Whether or not similar biomechanical property alterations occur in the cornea of myopic eyes is still under debate. Assessment of the corneal biomechanical properties may be a way to understand the possible etiology of high myopia development, which is of great importance in its prevention of development and progression [9].

The development of the Corneal Visualization Scheimpflug Technology (Corvis ST, Oculus, Wetzlar, Germany), a relatively new noncontact tonometer combined with an ultra-high-speed Scheimpflug camera, makes it possible for us to intuitively assess the corneal deformation response during an air impulse indentation in vivo. Hon [10] and Nemeth [11] have investigated the repeatability and reproducibility of parameters provided by the Corvis ST. There were several studies focusing on the potential differences in corneal biomechanical parameters between myopia and emmetropia. Lee et al. [12] indicated that there were significant differences in outward applanation velocity, peak distance and deformation amplitude in high myopia, low myopia and emmetropia. However, they did not take axial length (AL) into consideration, in fact that axial length elongation is the primary reason for progressive myopia [13]. Wang et al. [14] suggested that there were significant differences between emmetropia, moderate myopia and high myopia in terms of deformation amplitude, first- and second-applanation time, and radius at highest concavity. However, Matalia et al. [15] showed that corneal deformation parameters were unaffected by myopia. These studies did not investigate the correlation between the degree of myopia and the corneal biomechanical properties in severe myopia, which have an increased risk of myopic complications and deserve more attention.

The purpose of our study was to investigate the relationship between corneal biomechanical properties and the degree of myopia using the Corvis ST in a large sample taking into consideration AL and anterior segment parameters.

## Methods

Research was conducted in accordance with the Declaration of Helsinki and approved by the Ethics Committee of the Eye Hospital of Wenzhou Medical University. Informed consent was written from each subject before the examination. The study consisted of 265 eyes of 265 participants who presented to The Eye Hospital of Wenzhou Medical University seeking refractive surgery or for an optometric examination between January 2015 and December 2015. Subjects were excluded if they had a history of ocular surgery, any other ocular disease (e.g. corneal scaring, glaucoma and keratoconus) other than a refractive error. The exclusion criteria also included the presence of corticosteroid use, or systemic disease which could interfere with the eye such as diabetes mellitus and connective tissue diseases. Subjects who were pregnant or with astigmatism more than 3.00D were also excluded. Participants were required to stop wearing soft contact lenses at least 2 weeks and rigid lens at least 1 month before measurements were acquired. A clinician excluded glaucoma according IOP (intraocular pressure), optic nerve head assessment via fundus examination, mild keratoconus according to corneal topography. Based on spherical equivalent refraction (SE), subjects were divided into four groups: low myopia or control (SE from − 0.50 to − 3.00D), moderate myopia (SE from − 3.00 to − 6.00D), high myopia (SE from − 6.00 to − 10.00D) and severe myopia (SE greater than − 10.00D). In this study, all subjects had axial myopia.

All participants who met the inclusion criteria were selected randomly, with simple random sampling, and then received complete ophthalmic examinations including best visual acuity, subjective refraction, slit lamp microscope examination, fundus examination and measurements with the Pentacam HR (Oculus, Wetzlar, Germany), Lenstar LS900 (Haag-Streit AG, Koeniz, Switzerland), and Corvis ST (Oculus, Wetzlar, Germany). Only the right eye was measured to avoid the influence from high correlation between eyes. In cases were the right eye did not meet the inclusion criteria mentioned above, the left eye was included. All examinations were performed by an experienced ophthalmologist between 9:00 AM and 5:00 PM. The Corvis ST was the last device that was used, so as to eliminate the bias in corneal biomechanical variables caused by the air puff. All the instrument measurements were completed before pupil dilation. Three measurements with good quality were taken and the average value was used for statistical analysis. All corneal deformation images were reviewed immediately following each measurement and only images with an “OK” quality index were used in the analysis. If an “OK” was not achieved, the measurement acquisition was repeated until an “OK” was achieved. The Pentacam is a comprehensive anterior segment tomographer [16, 17] and the Lenstar LS900 is an optical biometer [18, 19]. The Corvis ST is a noncontact tonometer with an ultra-high-speed (4330 frames/s) Scheimpflug camera incorporated. It can dynamically record the deformation process of the cornea to an air impulse covering 8.0 mm. It automatically generates ten parameters: DA (the amount of corneal displacement at highest degree of concavity), A1-time and A2-time (time from start to the first and second applanation), A1-length and A2-length (length of flattened cornea at the first and second applanation), A1-velocity and A2-velocity (corneal velocity during the first and second applanation), HC-time (time from start to the highest concavity), PD (distance between the two peaks of the cornea at highest concavity), HC radius (radius of curvature at highest concavity). IOP values were also obtained with Corvis ST. Anterior segment parameters were measured with the Pentacam HR, including steepest keratometry (Ks), flattest keratometry (Kf), central corneal thickness (CCT), corneal volume (CV) and anterior chamber depth (ACD). AL was assessed with Lenstar LS900.

### Statistical analysis

Data were analyzed using SPSS software version 17.0. Normal distribution was assessed with the Kolmogorov–Smirnov test. Stepwise multivariable linear regression analysis was calculated to study the relationship between corneal biomechanical properties and ocular characteristics. A one-way analysis of variance (ANOVA) and a Fisher’s least significant difference *t*-test was used to analyze the differences in corneal biomechanical properties among the four groups. A *P*-value of < 0.05 was considered statistically significant.

## Results

Participant demographics are displayed in Table 1. This study included 265 eyes of 265 Chinese participants. Mean age was 27.02 ± 7.32 years (range: 18 to 52 years). Mean SE was − 7.29 ± 4.31D (range: − 0.63 to − 25.75D). Mean AL was 26.31 ± 1.82 mm (range: 21.87 to 31.94 mm). There were significant differences among groups in terms of refractive error and AL (*P* < 0.001). No significant differences were found between groups for CCT, IOP and CV (*P* > 0.05). Though there was a significant difference in age among four groups (*P* < 0.05), the difference was small. The one-sample Kolmogorov–Smirnov test showed that all data were normally distributed.

**Table 1:** Participant demographics

Parameters	Low myopia (n = 49)	Moderate myopia (n = 61)	High myopia (n = 91)	Severe myopia (n = 64)	*P-value*
Gender (male: female)	24:25	21:40	54:37	30:34	< 0.05
Age (years)	29.2 ± 8.85	24.58 ± 4.97	25.85 ± 5.98	29.34 ± 8.56	< 0.05
SE (D)	−1.92 ± 0.72	−4.64 ± 0.85	−7.84 ± 1.04	− 13.14 ± 3.27	< 0.001
Kf (D)	42.87 ± 1.83	42.49 ± 1.16	42.87 ± 1.31	43.34 ± 1.41	0.007
Ks (D)	43.85 ± 1.70	43.46 ± 1.23	44.02 ± 1.44	44.56 ± 1.41	< 0.001
CCT (μm)	537.22 ± 30.06	536.72 ± 32.95	538.92 ± 32.47	531.89 ± 31.72	0.599
CV (mm^3^)	60.12 ± 3.07	59.89 ± 3.49	60.57 ± 3.36	59.55 ± 3.42	0.291
ACD (mm)	3.12 ± 0.38	3.37 ± 0.31	3.25 ± 0.30	3.19 ± 0.31	< 0.001
AL (mm)	24.61 ± 1.28	25.42 ± 1.09	26.36 ± 1.13	28.39 ± 1.52	< 0.001
IOP (mmHg)	12.91 ± 2.21	13.07 ± 1.86	13.40 ± 2.39	13.67 ± 2.43	0.262

*SE* spherical equivalent, *AL* axial length, *Ks* steepest keratometry, *Kf* flattest keratometry, *CCT* central corneal thickness, *CV* corneal volume, *ACD* anterior chamber depth, *AL* Axial length, *IOP* intraocular pressure

Six of ten parameters (DA, A2-time, A2-velocity, A2-length, HC-time and HC-radius), were significantly different among the four groups (Table 2). DA in the low, moderate, high and severe myopia groups were 1.14 ± 0.11, 1.12 ± 0.09, 1.13 ± 0.10 and 1.18 ± 0.12 mm, respectively. SE was negatively correlated with AL (r = − 0.776, *P* < 0.001). DA in the severe myopia group was significantly longer than in the low, moderate and high myopia group (*P* = 0.04, *P* = 0.001, *P* = 0.006, respectively). A2-time in the low myopia group was significantly longer than the severe and high myopia group (*P* = 0.001, *P* = 0.023, respectively). A2-velocity in severe myopia group was significantly greater than in the low, moderate and high myopia group (*P* < 0.001, *P* < 0.001, *P* = 0.008, respectively). A2-length in the high and severe myopia groups were significantly smaller than in the other two groups. HC-time in the low myopia group was significantly greater than in the moderate, high and severe myopia groups (*P* = 0.02, *P* = 0.005, *P* < 0.001, respectively). HC-radius in the severe myopia group was significantly smaller than in the high myopia group. Table 3 described the coefficient (β) and *P* value for multivariate regression analysis. We did not find any relationship between PD and ocular parameters. AL was positively associated with DA whereas negatively associated with A1-velocity and A2-length. SE was positively associated with A2-time, HC-time and A2-velocity, whereas negatively associated with DA. IOP was positively associated with four corneal biomechanical parameters and negatively associated with three parameters. Age was positively associated with DA, A1-velocity and A2-time. CCT was positively associated with A1-length, HC radius and A2-length. ACD was negatively associated with A1-time, A2-velocity and HC-time.

**Table 2:** All the parameters acquired by the Corvis ST

Parameters	Low myopia (n = 49)	Moderate myopia (n = 61)	High myopia (n = 91)	Severe myopia (n = 64)	*P-value*
A1-time (m/s)	7.07 ± 0.21	7.07 ± 0.18	7.12 ± 0.24	7.14 ± 0.26	0.212
A1-length (mm)	1.76 ± 0.16	1.73 ± 0.12	1.73 ± 0.08	1.72 ± 0.09	0.293
A1-velocity (m/s)	0.15 ± 0.01	0.15 ± 0.01	0.15 ± 0.01	0.15 ± 0.01	0.602
A2-time (m/s)	21.93 ± 0.45	21.79 ± 0.36	21.76 ± 0.43	21.66 ± 0.42	0.011
A2-length (mm)	1.72 ± 0.28	1.71 ± 0.30	1.60 ± 0.25	1.55 ± 0.28	0.001
A2-velocity (m/s)	−0.38 ± 0.08	−0.40 ± 0.07	− 0.42 ± 0.07	− 0.46 ± 0.08	< 0.001
HC-time (m/s)	16.89 ± 0.38	16.69 ± 0.35	16.72 ± 0.32	16.59 ± 0.30	< 0.001
PD (mm)	4.16 ± 0.79	4.35 ± 0.66	4.23 ± 0.72	4.24 ± 0.79	0.619
HC radius (mm)	6.51 ± 0.79	6.63 ± 0.69	7.02 ± 0.86	6.52 ± 1.02	< 0.001
DA (mm)	1.14 ± 0.11	1.12 ± 0.09	1.13 ± 0.10	1.18 ± 0.12	0.007

A1-time and A2-time: time from start until the first and second applanation; A1-length and A2-length: length of flattened cornea at the first and second applanation; A1-velocity and A2-velocity: corneal velocity during the first and second applanation; HC-time: time from start until highest concavity is arrived; PD: distance between the two peaks of the cornea at highest concavity; HC radius: central curvature at highest concavity; DA: maximum deformation amplitude at highest concavity. One-way analysis of variance (ANOVA) and LSD-t test was used to analyze the differences of corneal biomechanical parameters between the four groups. *P* < 0.05 was considered statistically significant

**Table 3:** Stepwise multivariate regression model results

Parameters	Age (years)	SE (D)	Kf (D)	Ks(D)	CCT (μm)	CV (mm^3^)	ACD (mm)	AL (mm)	IOP (mmHg)
	β *P*	β *P*	β *P*	β *P*	β *P*	β *P*	β *P*	β *P*	β *P*
A1-time (m/s)							−0.020 0.025		0.989 < 0.001
A1-length (mm)					0.221 < 0.001				
A1-velocity (m/s)	0.133 0.022		0.222 < 0.001					−0.156 0.004	−0.373 < 0.001
A2-time (m/s)	0.111 0.005	0.129 0.001				0.169 < 0.001			−0.789 < 0.001
A2-length (mm)				−0.136 0.017	0.289 < 0.001			−0.248 < 0.001	0.177 0.004
A2-velocity (m/s)		0.408 < 0.001					−0.119 0.008		0.597 < 0.001
HC-time (m/s)		0.289 < 0.001	0.143 0.016				−0.234 < 0.001		
HC radius (mm)					0.207 0.001				0.355 < 0.001
DA (mm)	0.155 < 0.001	−0.119 0.039		0.084 0.026				0.151 0.008	−0.785 < 0.001

*SE* spherical equivalent, *Ks* steepest keratometry, *Kf* flattest keratometry, *CCT* central corneal thickness, *CV* corneal volume, *ACD* anterior chamber depth, *AL* Axial length, *IOP* intraocular pressure; A1-time and A2-time: time from start until the first and second applanation; A1-length and A2-length: length of flattened cornea at the first and second applanation; A1-velocity and A2-velocity: corneal velocity during the first and second applanation moment; HC-time: time from start until highest concavity is arrived; HC radius: central curvature at highest concavity; DA: maximum deformation amplitude at highest concavity

## Discussion

The present study aimed to assess the corneal biomechanics in eyes with different degrees of myopia, especially eyes with severe myopia, using the Corvis ST with a large sample size. In the current prospective study, we found that the severe myopia group had a significantly longer DA than the low, moderate and high myopia group. Moreover, DA was negatively correlated with SE (β = − 0.119, *P* = 0.039) and positively correlated with AL (β = 0.151, *P* = 0.008). DA is the deformation amplitude at highest concavity and is regarded as a surrogate for corneal stiffness [10, 14, 20]. A higher DA value would represent a softer cornea due to a lower resistance to deformation [20, 21]. Our findings showed that the higher the degree of myopia or the longer the axial length, the softer of the cornea, which is consistent with previous studies [12, 14, 21–24]. Miki et al. [24] have found similar trends but did not divide subjects into different groups according to SE or AL and had relatively few participants. In comparison, the present study included a large sample of subjects with severe myopia with rigorous screening criteria. Xiang et al. [25] suggested that eye elongates particularly fast before the onset of myopia. It is conceivable that changes in corneal and scleral biomechanics precede the occurrence of myopia. However, our data does not prove whether corneal biomechanical properties have changed with the onset of myopia. This would require a longitudinal study. In clinical practice, if a larger DA indicates a greater risk of axial elongation, preventive methods can be considered. Further, assessment of corneal biomechanics may also be crucial for surgical planning (and preoperative screening) to reduce the risk of corneal ectasia after refractive surgery.

A2-velocity in the severe myopia group was significantly greater than in the other three groups. A2-time in the high and moderate group was significantly shorter than in the low myopia group. The severe and high myopia groups had a smaller A2-length than other groups. These findings are not in line with those from Wang et al. [14], their study did not show the difference of A2-velocity between groups. Another study, consistent with our results, showed high myopia had greater A2-velocity compared to either low to moderate myopia or emmetropia using the Corvis ST [12]. A2-time and A2-velocity were positively correlated with SE, as per the ANOVA in the present study. A previous study found that A2-time may be an indicator of the total viscoelasticity of the cornea [26]. The higher the degree or the longer the AL of the myopic eye, the less time is needed to return to the origin after applanation, as its viscoelasticity increased. In a recent study [27], myopia had greater A2-velocity indicating that the cornea tends to be more deformable and softer than emmetropia and hyperopia in children. Therefore, A2-velocity may be another indicator of corneal elasticity, as Lee et al. [12], Miki et al. [24] and Wang et al. [28] suggested.

In accordance with a previous study by Wang et al. [14], we found that the HC radius was significantly smaller in eyes with severe myopia than high myopia. Frings et al. [29] demonstrated that in eyes following corneal refractive surgery, there was a tendency to have a smaller radius at highest concavity. That is, the softer the cornea, the easier it is to deform, resulting in a smaller HC-radius.

We found longer DA values with increasing age, in agreement with previous studies [14, 30]. However, others supported a view that the cornea becomes stiffer in elderly individuals because of more glycation-induced cross-linking [31, 32]. The relatively narrow range of age in this study may be a reason for the conflicting results. Further study is required to determine the influence of age on corneal biomechanics, controlling for other variables including SE. Our analysis showed a lower IOP was associated with less stiffness of the cornea with a larger DA (β = − 0.785, *P* < 0.001). This phenomenon is in accordance with previous studies [24]. Alonso et al. indicated that the variation in corneal deformation may depend on the IOP rather than simply affected by the corneal structure [33]. Tian et al. also found that DA was negatively correlated with IOP in both primary open-angle glaucoma (POAG) and normal eyes [34]. In other words, we should take the corneal biomechanical into consideration when it comes to the accuracy of IOP measurement.

It has been suggested that myopic eyes have a lower ocular rigidity than emmetropic eyes, [6, 7] and the diameter of collagen fiber bundles of sclera in highly myopic eyes is less and the sclera is significantly thinner [13, 35]. Less proteoglycan and glycosaminoglycan synthesis [36–38] and a reduction in the extracellular matrix [39] may explain the remodeling of the sclera during the development of myopia. In this way, the scleral mechanical properties weaken [35] whilst the deformability increases [40]. Since the corneal stroma is the continuation of the sclera, [35] the expansion of the sclera may result in the reduction of corneal stiffness. If a change in corneal biomechanical properties potentially indicate a risk for myopia development, such measurements may become clinically useful in the management of myopia. On the other hand, various studies have demonstrated that corneal refractive surgery may change corneal biomechanics, [21, 29, 41–43] which can lead to iatrogenic keratectasia [44]. Wang et al. [28] demonstrated that the postoperative refractive outcome of small-incision lenticule extraction (SMILE) can be predicted by using corneal topographic and biomechanical parameters (CTBPs). Therefore, a better understanding of the biomechanical characteristics of the cornea is of great importance in the field of corneal refractive surgery [45].

The main limitations of our study are as follows: first, we did not assess corneal deformation in emmetropia. Second, a longitudinal study is required to determine whether differences in corneal biomechanics is the consequence or the cause of myopia development.

## Conclusions

In conclusion, the myopic eyes with a greater degree and longer AL exhibited longer DA and A2-velocity, and less A2-time under stress, suggesting that the cornea in higher myopia tends to be more elastic and deformable. Further research is warranted in this field as this may have clinical significance the management of high myopia and also in refractive surgery.

## Data Availability

The datasets used and/or analyzed during the current study available from the corresponding author on reasonable request.

## Abbreviations

Corvis ST: Corneal visualization Scheimpflug technology
SE: Spherical equivalent
AL: Axial length
DA: Deformation amplitude
A2-time: Time from start until second applanation
A2-length: Length of flattened cornea at the second applanation
A2-velocity: Corneal velocity during the first and second applanation
HC-time: Time from start to highest concavity
HC radius: Central curvature at highest concavit
Ks: Steepest keratometry
Kf: Flattest keratometry
CCT: Central corneal thickness
CV: Corneal volume
ACD: Anterior chamber depth
IOP: Intraocular pressure
PD: Distance between the two peaks of the cornea at highest concavity
